# Genetic mapping of quantitative trait loci associated with nematode resistance in melon using genotyping-by-sequencing

**DOI:** 10.3389/fpls.2026.1744881

**Published:** 2026-03-20

**Authors:** Megha Sharma, Anroop Kaur, Sukhpreet Kaur Bhatia, Sat Pal Sharma, Sukhjeet Kaur, Deepika Narang, Nikkula Chandra Leela, Neha Kumari, Navraj Kaur Sarao

**Affiliations:** 1 School of Agricultural Biotechnology, Punjab Agricultural University, Ludhiana, Punjab, India; 2 School of Biosciences, Swami Rama Himalayan University, Dehradun, Uttarakhand, India; 3 Department of Vegetable Science, Punjab Agricultural University, Ludhiana, Punjab, India

**Keywords:** breeding, genotyping-by-sequencing, melon, quantitative trait loci, root-knot nematode

## Abstract

Muskmelon, a widely cultivated cucurbit crop valued for its delicious fruit, is severely threatened by different biotic stresses, including root**-**knot nematodes (RKNs). RKN, a soilborne pathogen belonging to the genus *Meloidogyne*, adversely affects crop yield, causing up to 30% damage in highly susceptible crops such as muskmelon. Developing resistant cultivars through breeding can be an effective approach to combat this disease. In the present study, 110 plants from the F_2:4_ mapping population were generated by crossing snapmelon accession “SM2012-1” (resistant parent) with melon cultivar “Punjab Sunehri” (susceptible parent) and used to map RKN resistance genes using genotyping-by-sequencing (GBS). Phenotyping was carried out by artificial screening of F_2:4_ plants against nematodes in a net house, in the rainy season, and in the spring season in 2023. Quantitative trait loci (QTL) analysis was conducted using WinQTL Cartographer, by integrating the high-density genetic linkage map (with an average marker density of 5.48 markers/Mb) and corresponding phenotypic data, which identified a total of nine QTLs associated with RKN resistance located on chromosomes 2, 8, 9, and 12. The QTLs were designated as *qRKN*_*Cm2.1*, *qRKN*_*Cm2.2*, *qRKN*_*Cm2.3*, *qRKN*_*Cm8.1, qRKN*_*Cm8.2*, *qRKN*_*Cm9.1*, *qRKN*_*Cm9.2*, *qRKN*_*Cm12.1*, and *qRKN*_*Cm12.2*, respectively. Among these, three QTLs (*qRKN*_*Cm8.1*, *qRKN*_*Cm12.1*, and *qRKN*_*Cm12.2*) were identified in the net house exhibiting phenotypic variance explained (PVE) that ranged from 10.1% to 26.8%. Two QTLs (*qRKN*_*Cm2.1* and *qRKN*_*Cm9.1*) were detected in the rainy season with a PVE of 8.2% to 8.3%, while four QTLs (*qRKN*_*Cm2.2*, *qRKN*_*Cm2.3*, *qRKN*_*Cm8.3*, and *qRKN*_*Cm9.2*) were identified in the spring season, with a PVE of 8.1% to 10.3%. The logarithm of odds (LOD) score for these QTLs ranged from 2.5 to 3.3. QTL mapping of RKN resistance carried out in the present study will provide linked molecular markers for marker-assisted selection (MAS), thereby providing a valuable resource for effective deployment of RKN resistance in melon breeding programs to developing elite resistant cultivars.

## 1 Introduction

Muskmelon (*Cucumis melo* L.) is an economically valuable and morphologically diverse warm-season vegetable crop that belongs to the family Cucurbitaceae (Jahan et al., 2024). It is composed of approximately 90 genera and 750 species globally (Langer and Hill, 1991). In terms of fruit production, melon ranked as the fourth most produced fruit crop worldwide after oranges, bananas, and grapes (Fatima et al., 2023). The total production of melons exceeds 28.30 million metric tons in over 100 countries (FAO, 2023, 2024). According to FAO, India is reported as the third largest producer of muskmelon with a 50, 000-hectare area and 1, 097, 000 MT production (FAO, 2019).

Muskmelon is highly prone to several types of pathogens such as root-knot nematodes (RKNs), *Fusarium* wilt, and begomovirus, which constantly threatens its sustainable production. RKNs are a soilborne pathogen caused by the genus *Meloidogyne*, which includes the three most prevalent species, i.e., *Meloidogyne incognita*, *Meloidogyne javanica*, and *Meloidogyne arenaria.* Among these, *M. incognita* is the most predominant species that infects members of cucurbitaceae, such as cucumber (*Cucumis sativus*), melon (*C. melo*), and watermelon (*Citrullus lanatus*) (Shahwar et al., 2024). RKNs resulted in a yield loss of more than 30% in melon as well as other highly vulnerable vegetable and fruit crops (Sikora and Fernandez, 2005). These nematodes cause gall formation on the tender roots of melon, thereby hindering the uptake of minerals and water, causing mechanical injuries. Infested plants exhibit several symptoms, including root swelling, chlorosis, stunted growth, leaf curling, daytime wilting, and overall decline (Basavaraj et al., 2024). Moreover, nematode-infected roots become highly vulnerable for invasion by other soilborne pathogens such as *Fusarium*, leading to complex disease interactions that are difficult to manage and can result in significant yield losses (Basavaraj et al., 2024; Dhami et al., 2024). Additionally, because of the endoparasitic nature of RKNs, they can survive in soil for many years, making the management more complex and challenging once they are established in agricultural fields (Basavaraj et al., 2025).

Host resistance is an effective and efficient method for the control of pathogens (Gordon and Martyn, 1997). However, cultivated cucurbits, particularly *C. melo* and *C. sativus*, exhibit high susceptibility to RKNs, and stable resistance in commercial cultivars remains limited (Sigüenza et al., 2005; Mukhtar et al., 2013). In contrast, resistance to *Meloidogyne* spp. has been reported in several wild *Cucumis* species, including *C. metuliferus, C. myriocarpus, C. moschata, C. postulatus, C. subsericeus*, and *C. zeyheri* (Pofu et al., 2011; Guan et al., 2014; Expósito et al., 2018). In addition to these, Indian snapmelon (*C. melo* var. *momordica*), locally known as “Phut”, is a valuable genetic resource due to its broad resistance to multiple biotic and abiotic stresses, including RKNs (Dhillon et al., 2014). The snapmelon accessions conserved at Punjab Agricultural University, Ludhiana (SM2012-12, SM2013-1, SM2013-2, SM2012-1, and SM2013-9), have been reported to possess resistance to RKNs and other diseases (Dhami et al., 2022; Patel et al., 2024; Dhami et al., 2024). Most screening studies have successfully identified resistant accessions; however, they have not yet established links between resistance traits and specific genomic regions using markers suitable for marker-assisted selection (MAS). The introgression of resistance genes from such sources into elite muskmelon cultivars through marker-assisted breeding offers a promising approach, particularly with the advent of high-throughput genotyping technologies that enable precise gene transfer with minimal linkage drag.

Genotyping-by-sequencing (GBS) is a robust and cost-effective method for generating high-density single-nucleotide polymorphism (SNP) markers and has been extensively applied for quantitative trait loci (QTL) mapping and MAS across diverse crops (Elshire et al., 2011; Semagn et al., 2014). The method employs a simple library construction protocol and effectively produces thousands of genome-wide markers (Elshire et al., 2011). Furthermore, the development of high-quality and high-density genetic maps at minimal cost can be facilitated through various bioinformatics pipelines, including imputation methods for raw data evaluation, SNP calling, and error correction (Spindel et al., 2013; Edae et al., 2015). The SNP-based GBS analysis has already been used in various crops for QTL mapping, genetic diversity analysis, genetic map construction, improving reference sequence, MAS, genomic selection, and allele mining (Semagn et al., 2014; Hyun et al., 2021; Kishore et al., 2021; Gebeyehu et al., 2025; Abdelhamid et al., 2026). Although resistance to RKNs has been identified in snapmelon (*C. melo* var. *momordica*), the underlying genetic architecture governing this resistance has not been adequately characterized. Keeping in view the importance of the crop, the present study aimed to dissect the inheritance of RKN resistance in muskmelon and identify associated QTLs using a reference-based GBS approach. The findings of this study will serve as a valuable tool for accelerating the breeding programs in melon.

## 2 Materials and methods

### 2.1 Experimental material

In the present study, the mapping population was developed by crossing two melon genotypes, Punjab Sunehri (PS) and Snapmelon (SM2012-1), at the Vegetable Research Farm, Department of Vegetable Science, Punjab Agricultural University, Ludhiana, Punjab. The university is located at 30°54′ N latitude and 75°48′ E longitude, at an altitude of 247 m above mean sea level. The soil at the research farm is sandy loam in texture. PS is a popular muskmelon variety among the state growers. It has a unique aroma, high TSS, and good transport ability but is susceptible to RKNs. SM2012-1 is a landrace accession adapted to Punjab state’s agroclimatic conditions, possessing resistance against the prevalent RKN*, M. incognita* populations (Dhami et al., 2024). The single-seed descent (SSD) method was used to produce F_2_-derived lines, viz., F_2:3_ and F_2:4_. In July 2021, the two parental lines (SM2012-1 × PS) were crossed to produce F_1_ plants. Selected F_1_ plants were subsequently self-pollinated under field conditions to generate the F_2_ generation. In July 2022, 300 F_2_ plants were grown under polyhouse conditions; however, only 167 plants survived to maturity. Subsequently, a single seed from each surviving F_2_ parent was used to advance the F_3_ generation. To ensure adequate plant stand, five to six seeds obtained from every single seed-derived plant were sown, of which only one plant was randomly retained and further advanced. Subsequently, one seed per plant was advanced in each successive generation without any phenotypic selection. Because of normal plant survival and handling losses, 110 F_2:4_ lines were finally obtained using the SSD method. Seedlings approximately 25–30 days old were transplanted into rows spaced 60 cm apart at 1.5 cm depth with 20 cm in-row spacing. The populations were grown under three environmental conditions, viz., spring, rainy, and net house during 2023, following standard agronomic practices.

### 2.2 Phenotypic screening

A total of 167 plants from the F_2_ generation, 150 plants from the F_2_:_3_ generation, and 110 plants from the F_2_:_4_ mapping population, along with both parental lines (PS and SM2012-1), were artificially screened for RKN resistance. A total of 110 F2:4 progenies were subsequently cultivated in triplicate using a randomized block design under three different environmental conditions in 2023. The first set of population was kept inside a poly-net house with controlled environmental conditions [25 ± 2°C temperature and 60%–70% relative humidity (RH)], providing optimal conditions for RKN to induce infection. The second set of population was screened during the rainy season (25–31°C, 40%–85% RH) and the third during the spring season of the same year (15–37 °C, 25%–90% RH). These different environmental conditions enabled comprehensive insights into disease dynamics while minimizing environmental variability in trait assessment.

**Figure 1:**
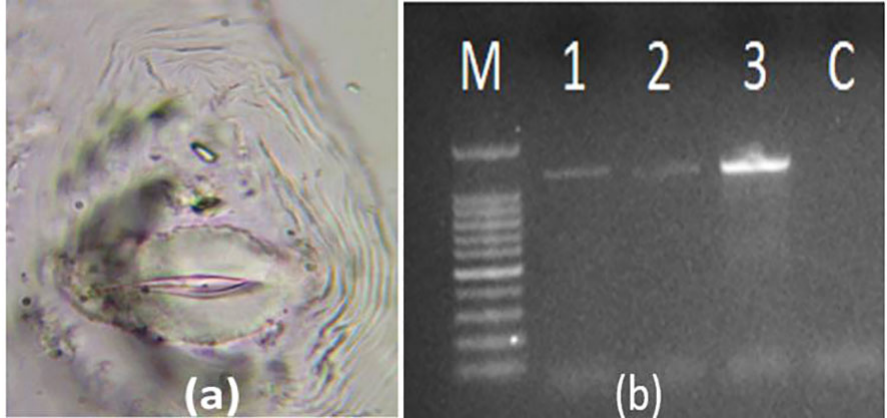
Perineal pattern of adult root-knot nematode females **(a)***Meloidogyne incognita*; **(b)** agarose gel (1%) showing amplification of 1, 200 bp with SCAR Finc/Rinc primer 1, M—marker/ladder (100 bp).

For screening of the F_2_ population, the seeds were directly sown in protrays filled with coco-peat, perlite mixture, and vermiculite (3:1:1) and inoculations were done using second-stage juveniles (infective stage) of *M. incognita.* The inoculum was taken from the pure culture of *M. incognita* maintained in pots on the susceptible brinjal cultivar “Punjab Sadabahar”. The culture isolate was confirmed by perineal pattern analysis of the mature females extracted from the raised culture as well as by polymerase chain reaction (PCR) amplification of the genomic DNA using *M. incognita*-specific Finc/Rinc (Zijlstra et al., 2000) primers (Kaur et al., 2016) (**Figure 1**). To prepare the inoculum, freshly hatched second-stage juveniles (J2) of *M. incognita* were collected using the Baermann funnel technique (Whitehead and Hemming, 1965). Infected roots were thoroughly washed, cut into 1- to 2-cm segments, and placed on double-layered tissue paper supported by a wire mesh. The mesh was positioned in a Baermann funnel filled with distilled water, ensuring the root material was just submerged. The setup was maintained at room temperature (25 ± 2 °C) for 24–48 h, allowing the active juveniles to migrate from the root tissues into the water. The resulting nematode suspension was collected from the base of the funnel and concentrated. The J2 populations were quantified using a stereomicroscope to prepare standard inoculum suspension of 1, 000 J2/mL.

Once the plants reached the three- to four-true-leaf stage, they were inoculated with freshly hatched second-stage juveniles of *M. incognita* at 1, 000 J2/plant. For screening of F_3_ and F_4_ populations, the seeds were directly sown in protrays filled with coco-peat, perlite mixture, and vermiculite (3:1:1), and once the plants reached the three- to four-true-leaf stage, they were transplanted into polybags (6-inch diameter) containing 1.25 kg of sterilized potting medium (soil + FYM in a 3:1 ratio). After 1 week of transplanting, as the plants were established in the polythene bags, RKN inoculations were performed with freshly hatched second-stage juveniles of *M. incognita* at 2, 500 J2/plant. The inoculations were done by making small holes near the root zone of each plant using a glass rod and pouring the nematode suspension around the roots. The plants were maintained properly and irrigated as per the crop requirement. The observations were recorded on number of galls per root system after 45 days of inoculations, and based on the number of galls per plant, the root galling index (RGI) was computed using a 0–5 scale given by Taylor and Sasser (1978), where RGI 0 = 0 galls/root, 1 = 1–2 galls/root, 2 = 3–10 galls/root, 3 = 11–30 galls/root, 4 = 31–100 galls/root, and 5 = more than 100 galls/root. Individual plant from each population was then classified as resistant or susceptible based on RGI, where RGI 0 = immune, 1 = resistant, 2 = moderately resistant, 3 = moderately susceptible, 4 = susceptible, and 5 = highly susceptible. As for the inheritance of resistance against RKN, *M. incognita* was computed by analyzing the phenotypic reaction of the F_2_ population and F_2:3_ progenies against *M. incognita* exhibited during the artificial screening.

### 2.3 Genomic DNA extraction, library construction, and sequencing

For genotyping, genomic DNA extraction was carried out from the young leaf tissues of both the parents (SM2012–1 and PS) and F_2:4_ populations using the modified CTAB protocol (Doyle and Doyle, 1990). DNA quality was assessed by electrophoresis on 0.8% agarose gel, and only samples showing intact, high-molecular-weight DNA without degradation were selected. DNA purity was evaluated using a NanoDrop™ 1000 spectrophotometer (Thermo Fisher Scientific, Wilmington, USA, and samples with an A260/280 ratio of 2.0 was retained). The quality of DNA was analyzed on 0.8% agarose gel electrophoresis, and the quantity was measured on a NanoDrop™ 1000 spectrophotometer (Thermo Fisher Scientific, Wilmington, USA). The high-quality DNA were then diluted to a final concentration of 100 ng/µL and selected for sequencing through GBS. Library construction and sequencing were outsourced to NGB Diagnostics (India). The restriction enzyme Ape-KI was employed to generate the library, and sequencing was performed on an Illumina HiSeqTM X10 platform (Illumina^®^ Inc., San Diego, CA, USA) with 159-bp paired-end (PE) reads.

### 2.4 Single-nucleotide polymorphism identification and linkage map construction

In our previous study (Kaur et al., 2025), a total of 3, 400 polymorphic SNPs were identified by using the Fast-GBS standard pipeline (Torkamaneh et al., 2017). FastQC software was used to check the quality control (QC) of both the parents and individuals (Andrews, 2010), followed by the removal of low-quality reads and adapters by using the Trimmomatic (version 0.39) software (ILLUMINACLIP: TruSeq3-PE_new.fa:2:30:10; LEADING:3; TRAILING:3; HEADCROP:10; SLIDINGWINDOW:4:15; MINLEN:36) (Bolger et al., 2014). The filtered reads were aligned to the reference genome sequence of melon (DHL92 v4; Garcia-Mas et al., 2012) obtained from the Melonomics database by performing the MEM algorithm of Burrow–Wheeler Aligner (BWA) (Li and Durbin, 2009), and alignment statistics were calculated for each sample. Samples with mapping rates below 70% were excluded, and only those with ≥73% alignment were retained for downstream analysis. Subsequently, sequence alignment map (SAM) files and binary alignment map (BAM) files were generated by using SAMtools. Variant calling was conducted by Genome Analysis Toolkit (GATK) HaplotypeCaller commands from the GATK suite, followed by SNP filtering via vcftools (variant calling format). Filtering parameters include a minimum read depth of 4, a minimum base quality of 20, a minor allele frequency (MAF) of ≤0.05, and the removal of missing data, heterozygous sites, and monomorphic SNP sites. Furthermore, the linkage map was constructed by using the Mapdisto (version 1.7.7) software (Lorieux, 2012). A total of 1, 962 SNPs were successfully mapped on the chromosomes after performing the chi-square (χ^2^) test, and unlinked and highly distorted markers were removed (Kaur et al., 2025). Consequently, these SNPs were grouped into 13 linkage groups (LGs) with a recombination frequency threshold of 0.3 and a logarithm of odds (LOD) value range of 3 to 6. The map distances in centimorgans (cM) were calculated using the Kosambi mapping function, and LGs were visualized with the software MapChart v2.2 (Voorrips, 2002).

### 2.5 Quantitative trait loci mapping

For QTL mapping, composite interval mapping (CIM) was performed using the Windows QTL Cartographer 2.5 software (Wang et al., 2012), with 1, 000 permutations at a 95% significance threshold, to identify QTLs associated with RKN resistance in the F_2:4_ population. The analysis was conducted using a window size of 10 cM to exclude cofactors closely linked to the test interval, and the genome was scanned at a walk speed of 1.0 cM. Marker cofactors were selected through forward–backward stepwise regression to control background genetic variation, while markers within the scanning window were excluded from cofactor selection. The significance threshold for QTL detection was determined using 1, 000 permutation tests at a 95% confidence level, and QTLs with LOD scores ≥2.5 (*p* < 0.05) were considered statistically significant. QTLs explaining ≥10% of the phenotypic variance explained (PVE) were classified as major QTLs, whereas those explaining <10% PVE were regarded as minor QTLs. The QTLs were named *qRKN_Cm2.1*, where “*qRKN*” indicates the QTL for resistance to RKN, “*Cm*” represents *C. melo*, and “*2.1*” denotes the first QTL identified on chromosome 2.

## 3 Results

### 3.1 Phenotypic screening and segregation analysis

The phenotypic evaluation of RKN was conducted under three environmental conditions by counting the number of galls formed in the roots of melon after 45 days of inoculation and indexing each plant on a 0–5 RGI scale (Taylor and Sasser, 1978). Gall formation represents the successful nematode penetration, establishment, and feeding site development within roots (Williamson and Gleason, 2003), while lesser or reduced galling reflects impaired nematode establishment and is a biologically meaningful indicator of host resistance (Williamson and Gleason, 2003; Moens et al., 2009).

**Figure 2:**
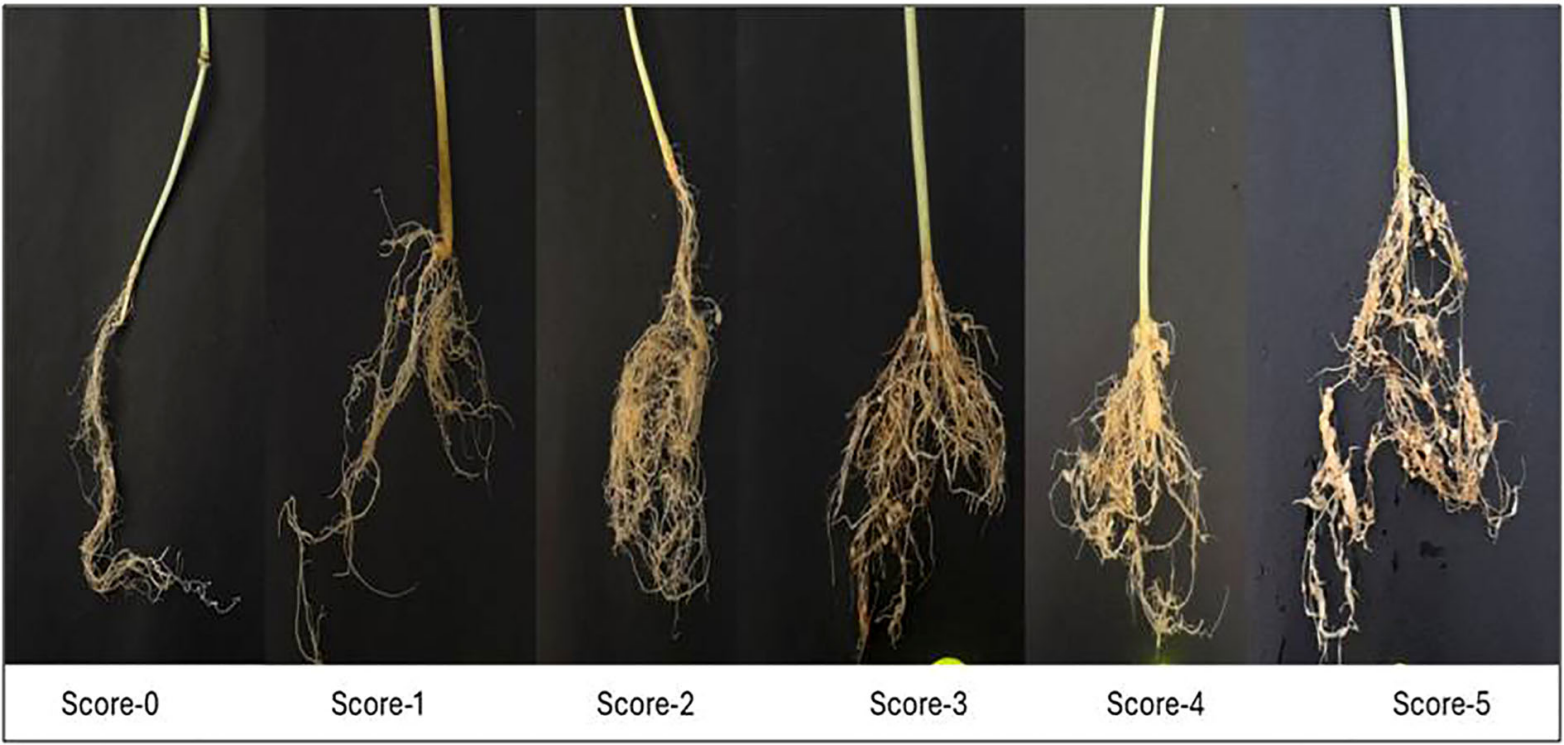
Gall formation in the roots of melon (0–5 scale used to compute the root galling index).

**Figure 3:**
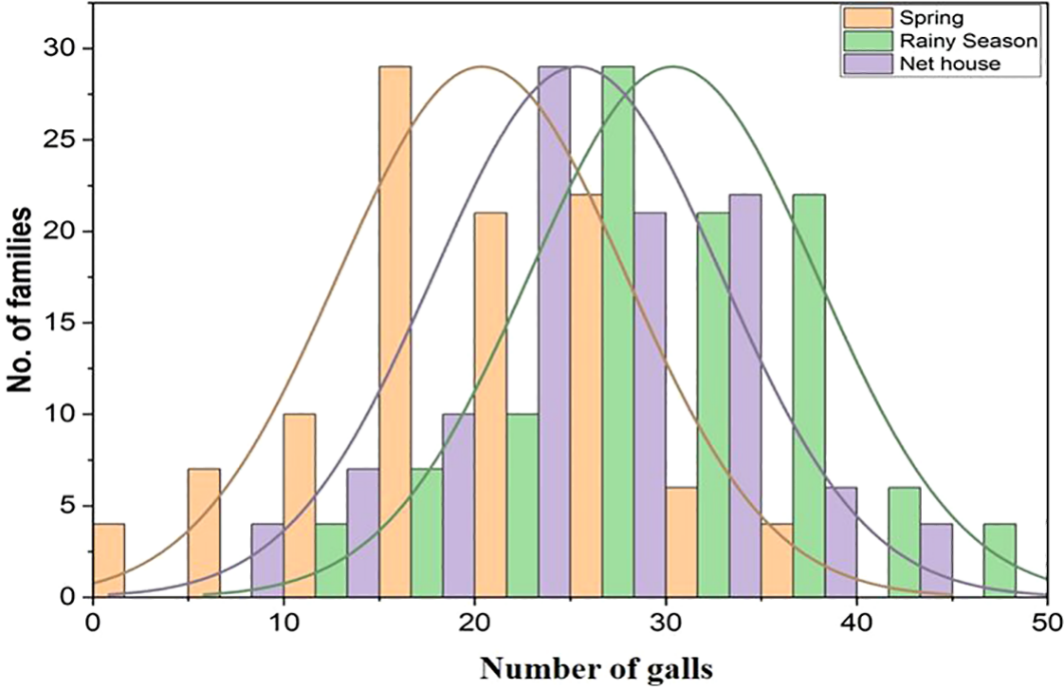
Frequency distribution of the disease in the spring season, rainy season, and net house.

In the present study, both parents showed contrasting phenotypic responses after inoculation with RKN. SM2012–1 was found to be moderately resistant with the presence of only two to five galls, while PS was found to be susceptible due to the occurrence of more than 50 galls per root system. The number of galls formed in the F_2:4_ melon roots varied from 7.41 to 41.62 (net house), from 12.54 to 46.80 (rainy season), and from 2.67 to 36.67 (spring season), followed by RGI scoring ranging from 1 to 5 based on a 0–5 scale (Taylor and Sasser, 1978) (**Figure 2**). The distribution of RKN resistance across all three environments showed skewness values close to zero (0.07–0.26), indicating nearly symmetrical distribution of the data. The kurtosis values were negative (−0.37 to −0.86), suggesting a slightly flatter distribution with wider dispersion of phenotypic values (**Table 1**). Continuous variation and a broad phenotypic range were observed in each environment. These results suggest that RKN resistance behaves as a quantitative trait likely controlled by multiple genes with additive effects and influenced by environmental conditions. The near-normal distribution of the data supports the suitability of QTL mapping for identifying genomic regions associated with the trait (**Figure 3**). The artificial screening against RKN was carried out in the F_2_ population and F_3_ progeny to know the genetic inheritance of RKN resistance, and the χ^2^ test was performed at the 5% level of significance (**Table 2**). A total of 167 F_2_ plants were screened, of which 139 were resistant and 28 were susceptible. The segregation showed significant deviation from the expected 3:1 ratio (χ² = 6.037, *p* < 0.05). Similarly, in the F_2_:_3_ generation, 150 progenies were segregated into 79 homozygous resistant, 51 heterozygous susceptible, and 20 homozygous susceptible lines, which also significantly deviated from the expected 1:2:1 ratio (χ² = 61.76, *p* < 0.05) (**Table 2**).

**Table 1:** Descriptive statistical result for the F_2:4_ population derived from the cross of Punjab Sunehri and SM2012-1.

Environment	Skewness	Kurtosis	Minimum	Maximum	Mean
Rainy season	0.07	−0.65	12.54	46.80	20.26
Spring season	0.26	−0.37	2.67	36.67	30.23
Net house	0.17	−0.86	7.41	41.62	25.62

**Table 2:** Segregation analysis of RKN resistance in the F_2_ and F_2:3_ populations.

Generation	Total	Disease reaction	Disease reaction	Disease reaction	χ^2^	χ^2^ (0.05)
		R	H	S		
F_2_	167 (Plants)	139 (R+H)		28	6.037* (3:1)	3.841
F_2:3_	150 (Progenies)	79	51	20	61.76* (1:2:1)	5.991

* symbol represented that the calculated value is greater than table.

### 3.2 Single-nucleotide polymorphism identification and linkage map construction

The high-density GBS-based linkage map generated by using SNP markers for mapping the FW resistance gene (Kaur et al., 2025) was used to map the RKN-resistant gene in melon. A total of 1, 962 SNPs were mapped on 13 LGs with a mapping efficiency of ~57.71%. The cumulative length of the linkage map was 5, 865.0 cM with an average of ~451.92 cM per LG. The length of LGs ranged from 252.10 to 635.10 cM, comprising 78 to 379 SNPs evenly distributed across the map, with a maximum spacing of 16.85 cM between the SNPs and an average distance of 3.47 cM.

### 3.3 Quantitative trait loci analysis

**Figure 4:**
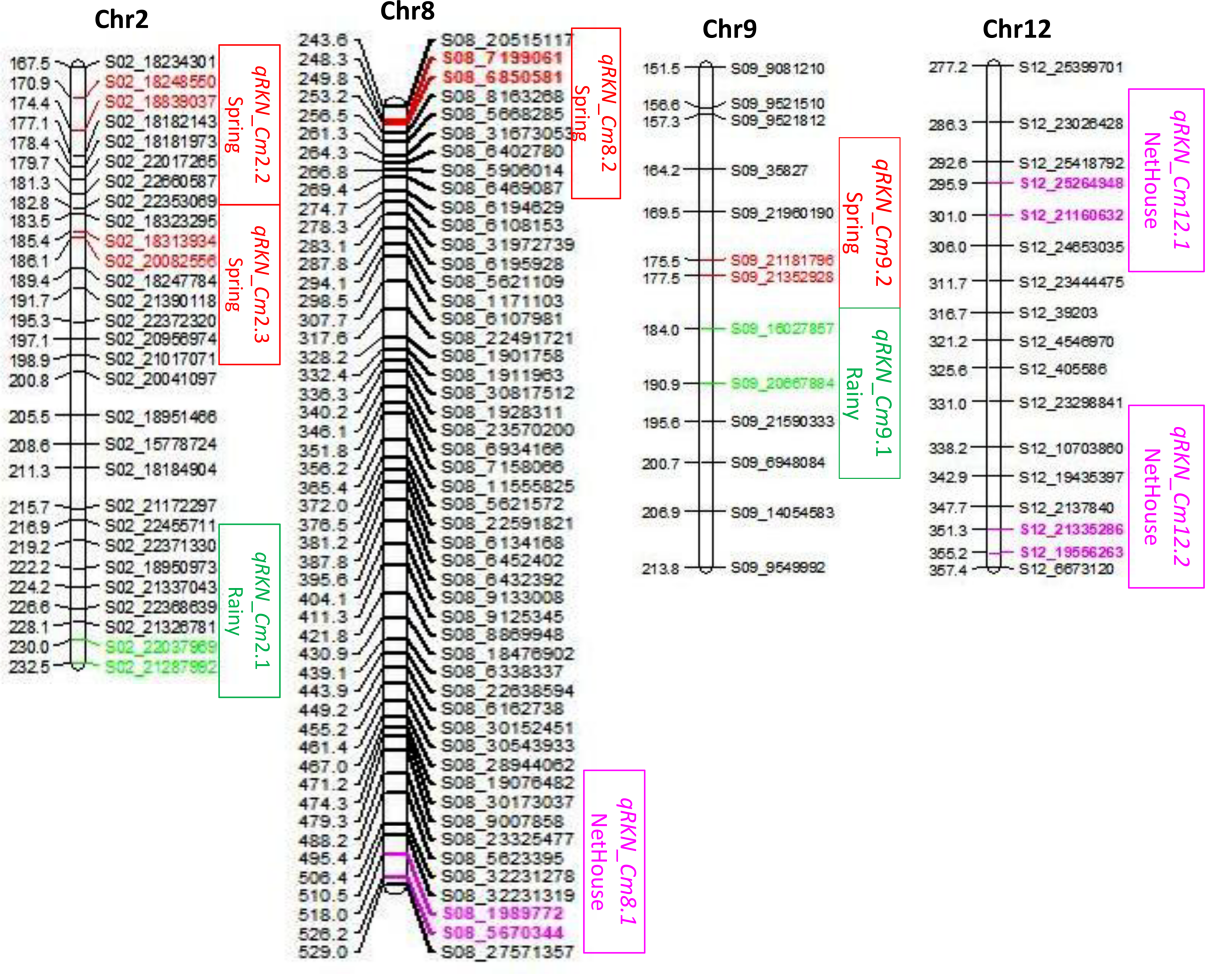
Representation of identified QTLs on chromosomes 2, 8, 9 and 12. QTLs identified in the net house, rainy season, and spring season are colored pink, green, and red, respectively.

Genotypic data along with their respective phenotypic data of F_2:4_ populations were used for the identification of QTLs associated with RKN resistance in melon (**Table 3**). A total of nine QTLs were identified on chromosomes 2, 8, 9, and 12. The QTLs designated as *qRKN*_*Cm8.1*, *qRKN*_*Cm12.1*, and *qRKN*_*Cm12.2* were identified under net house conditions. The QTL *qRKN_Cm8.1* linked to chromosome 8 accounted for 10.1% of the PVE, with a LOD score of 3.0 and an additive effect of −0.11. It was positioned at 526.2 cM, flanked by the markers S08_1989772 and S08_5670344, spanning a genetic interval of 8.17 cM. Two additional QTLs, *qRKN_Cm12.1* and *qRKN_Cm12.2*, were identified on chromosome 12, positioned at 295.9 and 355.1 cM, respectively. These QTLs explained 26.8% and 16.9% of the PVE, with LOD scores ≥2.5 and additive effects of 0.49 and 0.67, respectively. During the rainy season, two QTLs, i.e., *qRKN*_*Cm2.1* and *qRKN*_*Cm9.1*, were identified on chromosomes 2 and 9, contributing 8.2% and 8.3% to the PVE, respectively. These QTLs were positioned at 229.9 and 184.0 cM between the flanking markers S02_22037969-S02_21287992 and S09_16027857-S09_20667884 with a LOD score of 3.1 and 2.5 and a genetic interval of 2.57 and 6.87 cM, respectively. Similarly, during the spring season, a total of four QTLs—*qRKN*_*Cm*2.2, *qRKN*_*Cm*2.3, *qRKN*_*Cm*8.2, and *qRKN*_*Cm*9.2—were mapped on chromosomes 2, 8, and 9, with PVE ranging from 8.1% to 10.3% and LOD score ranging from 2.5 to 3.3. Overall, the cumulative PVE of the detected QTLs across environments ranged from 16.5% (rainy) to 53.8% (net house), indicating that RKN resistance is governed by both major and minor effect loci. **Figure 4** illustrates the identified QTLs on the genetic map, with different colors used to distinguish their associations across the three environments. The genetic positions of these QTLs were on 174.3, 185.4, 248.3, and 175.4 cM, respectively, and exhibited genetic intervals of 3.48, 0.67, 1.49, and 2.00 cM. QTLs on chromosome 8 were identified in both the net house and spring seasons, although the positions of the flanking markers differed. Likewise, QTLs on chromosomes 2 and 9 were common to the rainy and spring seasons, but their flanking marker positions varied between the seasons. **Figure 4** shows the identified QTLs on the genetic map, with different colors used to distinguish their associations across the three environments.

**Table 3:** Quantitative trait locus identified for RKN resistance in melon.

Trait	Environment	QTL	LG	Chr	Position	Marker interval	Interval (cM)	LOD score	PVE%	Additive
RKN	Net house	*qRKN_Cm8.1*	9	8	526.2	S08_1989772-S08_5670344	8.17	3.0	10.1	−0.11
RKN		*qRKN_Cm12.1*	13	12	295.9	S12_25264948-S12_21160632	5.04	2.8	26.8	0.49
RKN		*qRKN_Cm12.2*	13	12	355.1	S12_19556263-S12_21335286	3.89	2.5	16.9	0.67
RKN	Rainy	*qRKN*_*Cm*2.1	2	2	229.9	S02_22037969-S02_21287992	2.57	3.1	8.2	0.11
RKN		*qRKN*_*Cm*9.1	10	9	184.0	S09_16027857-S09_20667884	6.87	2.5	8.3	−0.35
RKN	Spring	*qRKN*_*Cm*2.2	2	2	174.3	S02_18839037-S02_18248550	3.48	2.6	9.2	−0.18
RKN		*qRKN*_*Cm*2.3	2	2	185.4	S02_18313934-S02_20082556	0.67	2.5	8.1	−0.19
RKN		*qRKN*_*Cm*8.2	9	8	248.3	S08_7199061-S08_6850581	1.49	3.3	8.1	0.32
RKN		*qRKN*_*Cm*9.2	10	9	175.4	S09_21181796-S09_21352928	2.00	2.5	10.3	0.31

Moreover, to evaluate seasonal stability, QTL peak positions detected in different environments were compared based on their chromosome number and genetic distance. On chromosome 9, the QTLs identified in the rainy season (184.0 cM) and spring season (175.4 cM) were only 8.6 cM apart. This close distance suggests that they may represent the same genomic region, indicating partial stability across seasons. However, the additive effects were in opposite directions in the two seasons, showing that environmental conditions may influence gene expression. In contrast, QTLs detected on chromosomes 2 and 8 were located far apart (40–270 cM) in different seasons. This large distance indicates that they are different genomic regions and are strongly influenced by environmental conditions. Overall, these findings suggest that resistance to RKN in melon is mainly controlled by season-specific QTLs, with only limited stability across environments.

## 4 Discussion

RKNs are a significant constraint in crop production, causing substantial losses in both yield and quality and also showing remarkable pathogenic potential on melon (Shahwar et al., 2024). Most cultivated cucurbit species are susceptible to RKNs, and it is extremely rare to obtain complete resistance in cultivated cucurbits, particularly *C. melo* and *C. sativus* (Sigüenza et al., 2005; Mukhtar et al., 2013). In addition, the nematodes are difficult to control using a single management strategy due to their wide host range and high fecundity. With increasing restrictions on chemical use because of environmental and health concerns, natural host resistance has become an effective, sustainable, and economical approach to integrated nematode management. Therefore, identification of resistance sources and understanding their genetic control through heritability studies are essential for breeding nematode-resistant cultivars. However, selection outcomes often vary due to the complex nature of plant–nematode interactions. de Lima et al. (2026) documented the genetic analyses of melon germplasm for RKN responses and estimation of genetic parameters for resistance using restricted maximum likelihood (REML) and predicting genotypic values through best linear unbiased prediction (BLUP). To date, no well-established QTLs conferring resistance to *Meloidogyne* spp. have been reliably mapped in *C. melo*. Therefore, mapping of RKN resistance genes in melon is crucial for strengthening the resistance breeding program and the development of nematode-resistant varieties.

Snapmelon has been reported to acquire resistance or tolerance to a range of biotic stresses, including RKNs (Dhillon et al., 2014). In the present study, snapmelon accession SM2012–1 was used as a resistant parent, while PS was used as a susceptible parent after screening them against nematode resistance. These findings are consistent with previous screening studies where SM2012–1 had shown moderate resistance to nematodes (*M. incognita*) while PS was found to be susceptible to the same RKN species (Dhami et al., 2024). Furthermore, the gene inheritance analysis of the F_2_ population and the F_2:3_ progenies revealed χ^2^ values significantly higher than the table value (**Table 2**). This deviation from the expected Mendelian ratio suggests that the resistance to *M. incognita* in muskmelon is governed by multiple genes. Similar findings were reported by Candido et al. (2017), who studied the inheritance pattern of RKN resistance in an F_2_ population derived from a cross between JAB 20 (*C. melo* var. cantalupensis Naud.) and “Gaúcho Redondo” cultivar (*C. melo* var. reticulatus Naud.), and their results also revealed the polygenic nature of RKN resistance, although several studies on different crops revealed contrasting inheritance patterns of RKN resistance. For instance, in sweet potato (Obata et al., 2022) and cowpea (Ndeve et al., 2019), the resistance against *M. incognita* is found to be governed by two genes, whereas monogenic resistance has been reported in grapes (Smith et al., 2018), capsicum (Changkwian et al., 2019), and tomato (Devran et al., 2023). However, studies on the inheritance of RKN resistance in melon are still limited, and the underlying genetic mechanism is not fully understood. Therefore, integrating different molecular tools and advanced breeding approaches can pave the way towards the identification of different RKN strains and the development of effective, sustainable management strategies. Furthermore, GBS analysis was performed in F_2:4_ populations to identify the RKN-resistant gene in melon. The number of SNPs identified in the present study varied from previous studies, which can be attributed to several factors, including the origin of germplasm, sequencing methods used, software used for variant calling, and filtering criteria. A total of 1, 962 SNPs were identified and mapped on the 13 LGs, which was comparable to the previous studies where 824 (Pereira et al., 2018), 1, 837 (Nimmakayala et al., 2016), and 2, 493 (Chang et al., 2017) SNPs were mapped while constructing the linkage map in their respective generated melon populations. These variations in the number of SNPs were due to the utilization of different melon accessions and differences in traits of interest. This approach has already been used in melon to characterize the different accessions of melon (Nimmakayala et al., 2016; Gur et al., 2017; Pavan et al., 2017), bi-parental populations (Nimmakayala et al., 2016; Chang et al., 2017; Pereira et al., 2018), and genetic diversity and population structure studies (Pavan et al., 2017). In addition, the GBS approach has already been employed for the development of linkage maps and conducting QTL mapping in several cucurbits, including bitter gourd, bottle gourd, pumpkin, squash, muskmelon, and watermelon (Rao et al., 2018; Wu et al., 2017; Zhang et al., 2015; Chang et al., 2017; Daley et al., 2017; Montero-Pau et al., 2017; Branham et al., 2018; Meru and McGregor, 2016). Genotypic data including genetic distances between the markers along with phenotypic data for RKN in three distinct environments were utilized for QTL mapping using the CIM of Windows QTL Cartographer 2.5 (Wang et al., 2012).

In the present study, a total of nine QTLs were identified on different chromosomes. The QTLs on chromosomes 8 and 12 of net house and chromosome 9 of spring season, i.e., *qRKN*_*Cm8.1, qRKN*_*Cm12.1*, *qRKN*_*Cm12.2*, and *qRKN*_*Cm9.2*, are considered as major QTLs as they had higher PVE values, ranging from 10% to 26%, while QTLs on chromosomes 2 and 9 of the rainy season and chromosomes 2 and 8 of spring season, i.e., *qRKN*_*Cm2.1, qRKN*_*Cm9.1*, *qRKN*_*Cm2.2, qRKN*_*Cm2.3*, and *qRKN*_*Cm8.2*, were considered as minor QTLs as they exhibited PVE value <10% (**Table 3**). The seasonal variation observed in the QTL position indicates the strong interaction between the environmental factors and genotype (G × E), suggesting that environmental factors influence the expression of RKN resistance. Major QTLs typically have large, consistent effects on trait variation, while minor QTLs contribute smaller, often environment-dependent effects. Furthermore, the five QTLs (*qRKN*_*Cm2.1, qRKN*_*Cm12.1*, *qRKN*_*Cm12.2*, *qRKN*_*Cm8.2*, and *qRKN*_*Cm9.2*) showed positive additive effects, signifying that the susceptible parent (PS) contributes to the alleles, whereas four QTLs (*qRKN*_*Cm8.1, qRKN*_*Cm9.1*, *qRKN*_*Cm2.2*, and *qRKN*_*Cm2.3*) showed negative additive effects, indicating the contribution of allele from the resistant parent (SM2012-1), further highlighting the quantitative and environment-responsive nature of RKN resistance. QTL mapping studies for RKN resistance in melon are still limited; however, previous studies have successfully identified QTLs linked to *Fusarium* wilt resistance in melon using bi-parental populations (Ren et al., 2015; Meru and McGregor, 2016; Branham et al., 2018, 2020). The moderate population size and marker density in the present study may have limited the detection of minor-effect QTLs and reduced mapping resolution. Additionally, environmental variability may have influenced phenotypic expression, highlighting the need for multi-environment validation. However, the identification of genetic regions associated with RKN resistance can provide a valuable foundation for future studies in melon breeding programs.

## 5 Conclusions

This study successfully identified nine QTLs associated with RKN resistance on chromosomes 2, 8, 9, and 12. The information generated on these QTLs is valuable for fine-mapping and MAS, thereby improving melon breeding programs aimed to develop RKN-resistant genotypes. However, the polygenic nature of resistance, marker limitations, and environmental variability may limit the selection accuracy. Therefore, integrating the MAS with genomic selection can improve selection efficiency, accelerate genetic gains, and enhance crop resilience in breeding programs.

## Data Availability

The data for this article is available at repository NCBI, accession number: PRJNA1122332.
